# Refining Patient Selection Criteria for LV-Only Fusion Pacing in Cardiac Resynchronization Therapy: A Systematic Review

**DOI:** 10.3390/jcm14144853

**Published:** 2025-07-08

**Authors:** Adelina Andreea Faur-Grigori, Cristina Văcărescu, Samuel Nistor, Silvia Ana Luca, Cirin Liviu, Simina Crișan, Constantin-Tudor Luca, Radu-Gabriel Vătășescu, Dragoș Cozma

**Affiliations:** 1 Doctoral School, “Victor Babeș” University of Medicine and Pharmacy, 300041 Timisoara, Romania; andreea.faur@umft.ro (A.A.F.-G.); silvia.luca@umft.ro (S.A.L.); liviu.cirin@umft.ro (C.L.); 2 Institute of Cardiovascular Diseases Timisoara, 300310 Timisoara, Romania; samuel.nistor@umft.ro (S.N.); simina.crisan@umft.ro (S.C.); constantin.luca@umft.ro (C.-T.L.); dragos.cozma@umft.ro (D.C.); 3 Research Center of the Institute of Cardiovascular Diseases Timisoara, 300310 Timisoara, Romania; 4 Department of Cardiology, “Victor Babeș” University of Medicine and Pharmacy, 300041 Timisoara, Romania; 5 Center for Modeling Biological Systems and Data Analysis, “Victor Babeș” University of Medicine and Pharmacy, 300041 Timisoara, Romania; 6 Department of Cardiology, Faculty of Medicine, Carol Davila University of Medicine and Pharmacy, 050474 Bucharest, Romania; radu.vatasescu@umfcd.com; 7 Department of Cardiology, Emergency Clinical Hospital of Bucharest, 014461 Bucharest, Romania

**Keywords:** cardiac resynchronization therapy, CRT, biventricular pacing, LV-only, univentricular pacing, fusion, selection criteria, RA/LV dual-chamber device, physiologic pacing, responder

## Abstract

**Objectives:** This review aims to systematically evaluate the clinical outcomes of left ventricle-only fusion pacing (LV-only fCRTp) and identify evidence-based selection criteria that may optimize patient response and long-term therapeutic benefit. **Background**: Cardiac resynchronization therapy (CRT) is traditionally associated with biventricular pacing (BiVp). However, approximately 20–40% of patients seem to remain non-responders to this therapy. LV-only fCRTp offers a more physiological alternative by combining left ventricular epicardial pacing with the intrinsic ventricular activation wavefront. Beyond optimization strategies, the observed variability in response highlights the need for better patient selection in order to fully unlock its therapeutic potential. **Methods**: A systematic literature search was conducted in PubMed and Cochrane Library for original articles published up to April 2025, following PRISMA 2020 guidelines. The search focused on LV-only fCRTp performed either through standard RA/LV/RV biventricular devices or RA/LV dual-chamber systems. **Results**: Twenty-seven studies met the inclusion criteria. Among these, 17 studies obtained LV-only fCRTp using biventricular devices, and 10 were considered true LV-only fCRTp using RA/LV dual-chamber devices. Standard and specific selection criteria were used to qualify patients for LV-only fCRTp. Preserved atrioventricular conduction, ischemic cardiomyopathy, arrhythmic risk stratification, and the management of supraventricular arrhythmias were common overlapping parameters among studies with high variability, highlighting their potential role in response. RA/LV devices yielded consistent clinical benefits and low complication rates, particularly in nonischemic patients with stable AV conduction and low arrhythmic risk, while having a lower financial burden. **Conclusions**: Beyond guideline recommendations for CRT, this review identifies supplementary selection criteria that could further influence the effectiveness and stability of fusion pacing.

## 1. Introduction

Traditional cardiac resynchronization therapy (CRT) relies on biventricular pacing (BiVp), where both the right and left ventricles are simultaneously paced to restore synchronized contraction. However, the clinical benefits of CRT are not consistent for all patients, with response rates varying significantly. Studies indicate that over 30% of patients fail to achieve a favorable response [1,2]. The primary ongoing concern regarding non-response remains the detrimental impact of chronic right ventricular (RV) pacing [3,4,5]. Furthermore, Varma et al. demonstrated that patients with chronic heart failure (HF) and left bundle branch block (LBBB) generally maintain a preserved conduction through the right-sided His-Purkinje system, making the presence and functional necessity of a right ventricular (RV) lead questionable in this population [6]. Over the past two decades, numerous strategies have been explored to optimize CRT and enhance patient response. Since the beginning, left ventricle-only fusion pacing (LV-only f-CRT) has emerged as a promising resynchronization strategy. The fundamental mechanism that makes LV-only fCRTp more physiological is the preservation of intrinsic RV conduction, allowing for a more natural and synchronous ventricular activation [7,8].

In the RAFT trial, patients with chronic RV pacing did not achieve the same clinical outcomes after CRT as those with intrinsic LBBB. This discrepancy was attributed to fundamental differences in the electrical and mechanical activation pattern [9]. In conventional BiVp, both the RV and left ventricle (LV) are paced simultaneously, bypassing the intrinsic His-Purkinje conduction system. This results in a non-physiological activation pattern, where the RV is typically activated from the apical or septal pacing site via slow myocardial conduction. In contrast, LV pacing from the lateral or posterolateral coronary vein generates epi-endocardial electrical wavefront propagation. Despite aiming to achieve resynchronization, septal contraction is disrupted, leading to a paradoxical septal wall motion. In fact, septal contraction contributes approximately 30–40% of total LV mechanical efficiency [10].

In contrast to BiVp, LV-only fCRTp preserves intrinsic conduction via the right bundle branch (RBB) through optimal atrioventricular (AV) interval programming. This enables an ideal fusion between the intrinsic and LV-paced activation wavefronts. The benefit of this approach is its ability to facilitate multisite activation of the RV and interventricular septum, as impulses traveling through the fast-conducting Purkinje system emerge at multiple endocardial sites. This maintains better overall intraventricular synchrony and minimizes the risk of pacing-induced cardiomyopathy [11].

Several studies emphasize the importance of careful patient selection for LV-only fCRTp and the necessity of fusion optimization to achieve maximal clinical benefits. It has also been highlighted that trials comparing conventional BiVp to LV-only fCRTp often lacked standardized selection criteria and systematic optimization protocols [12]. Therefore, this review aims to conduct a comprehensive analysis of the existing literature on inclusion criteria and clinical outcomes related to LV-only fCRTp, to establish an evidence-based model for patient selection.

## 2. Materials and Methods

This systematic review was reported according to the PRISMA 2020 statement guidelines and was registered in PROSPERO (https://www.crd.york.ac.uk/PROSPERO/; (registration ID: CRD420251034452), accessed on 25 April 2025).

**Eligibility Criteria**

The PICO framework was used to develop a set of criteria for refining study selection:

– P (Population): adult HF patients, LVEF ≤ 35%, symptomatic despite GDMT, and with evidence of electrical dyssynchrony (LBBB with QRS duration ≥ 120 ms);
– I (Intervention): LV-only fCRTp achieved either through RA/LV/RV biventricular devices or RA/LV dual-chamber devices (without a right ventricular lead);
– C (Comparison): conventional BiVp;
– O (Outcomes): all-cause and cardiovascular mortality, functional response to CRT, echocardiographic reverse remodeling, super-responder rates, heart failure-related hospitalizations, incidence of arrhythmic events or need for device upgrade to either CRT-D or BiVp in LV-only fCRTp groups, and device longevity.

Eligible articles were required to report outcomes in patients who underwent LV-only fCRTp. Comparative studies with conventional BiVp were also considered, provided that outcomes related to LV-only fCRTp were reported.

Articles that did not report on patients with a clear CRT indication or without a clearly defined LV-only population, or those that did not provide clinical, echocardiographic, arrhythmic, or device-related outcomes related to LV-only fCRTp, were excluded from the analysis. Studies limited to acute response or peri-procedural endpoints without a minimum follow-up of three months were not considered. Unpublished data, grey literature, and conference abstracts were excluded.

**Information sources and search strategy**

The search was conducted in MEDLINE via PubMed and also the Cochrane Library, retrieving articles published in English up to 20 April 2025. The search strategy included combinations of the following keywords: cardiac resynchronization, biventricular pacing, fusion, LV only, left ventricular pacing, LV pacing, left ventricular only, univentricular pacing, left univentricular, LUV, and LV CRT. Complete search strings can be consulted in the Supplementary Materials (S1).

**Selection process and data collection**

**Figure 1:**
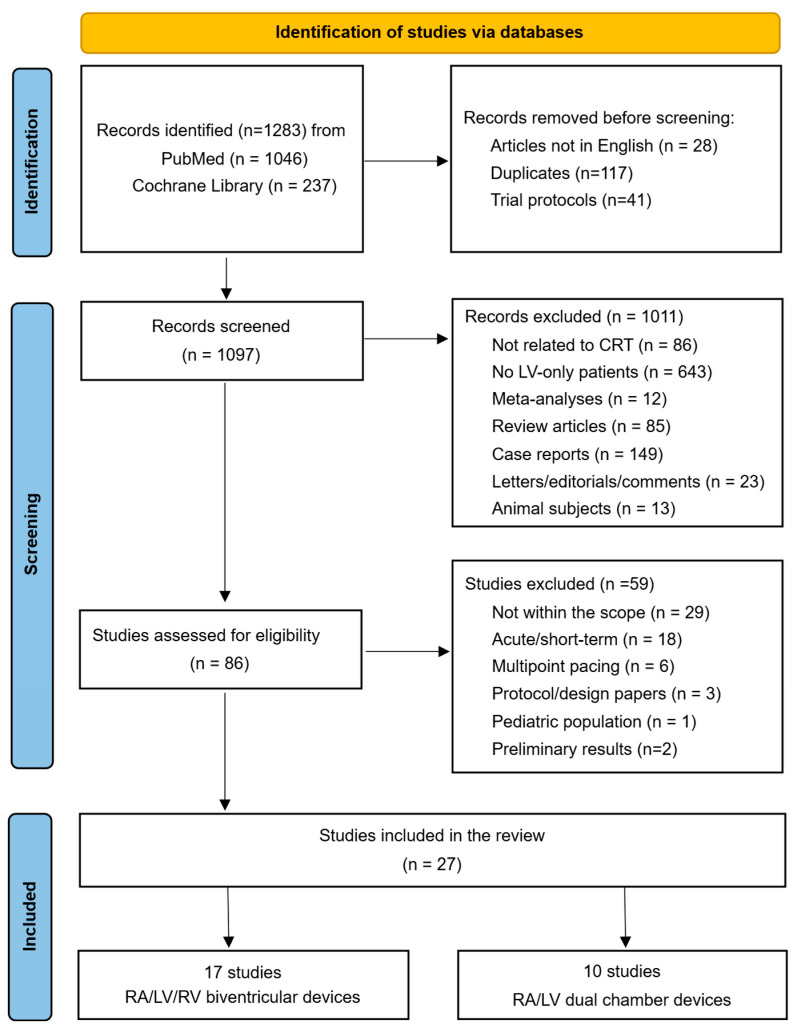
PRISMA 2020 flow diagram of study selection process.

Study screening and selection were carried out independently by two reviewers in a blinded manner to reduce selection bias. Titles and abstracts were screened initially, followed by a full-text analysis of potentially eligible articles. Any disagreements were resolved through discussion until consensus was reached. The complete study selection process is presented in the PRISMA flow diagram (Figure 1).

**Data Items**

Investigated outcomes were previously defined in the PICO framework. Two reviewers independently extracted data using a standardized spreadsheet. The following study-level variables were collected: first author, year, study name, design, number of patients, inclusion criteria, exclusion criteria for LV-only pacing, type of device used, fusion optimization method (FOI), and duration of follow-up. No assumptions were made for missing data, and unreported items were noted accordingly.

**Risk of Bias Assessment**

Risk of bias was assessed according to study design. For individually randomized parallel-group trials, the standard version of the Cochrane Risk of Bias Tool 2.0 (RoB 2) was used. For crossover randomized trials, the dedicated RoB 2.0 tool for crossover designs was applied. For non-randomized studies, the Methodological Index for Non-Randomized Studies (MINORS) was used. All assessments were performed independently by two reviewers, cross-checked, and summarized in Appendix A.

**Synthesis Methods**

Study characteristics and outcomes were presented narratively and summarized in both tabular and graphical formats. No meta-analysis was performed due to heterogeneity regarding inclusion criteria, designs, endpoints, and pacing protocols.

## 3. Results

**Figure 2:**
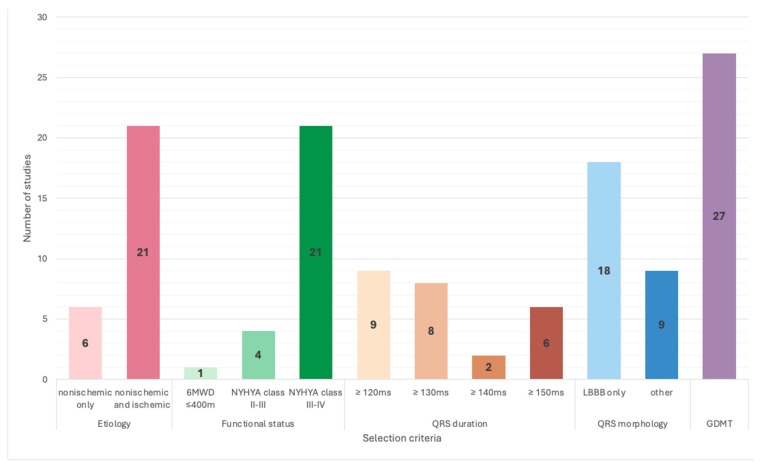
Distribution of common standard selection criteria for LV-only fCRTp.

A total of 27 studies were included in this systematic review, according to the selection process detailed in the PRISMA 2020 flow diagram (Figure 1). Of these, 17 studies directly compared LV-only fCRTp with BiVp (Table 1) while the remaining 10 studies explored true LV-only fCRTp using RA/LV dual-chamber devices without an RV lead (Table 2). Across all studies, patients were first selected using evidence-based guideline recommendations (Figure 2). All patients had guideline-directed medical therapy (GDMT) in common for at least 3 months before implantation and an LVEF under 35%. A QRS duration ≥ 120 ms was a standard requirement. However, minimum QRS thresholds varied among studies, with some using stricter cutoffs of ≥130 ms, ≥140 ms, or even ≥150 ms. Notably, 18 studies focused on including patients with LBBB, while some accepted other QRS morphologies. Functional status was also variably defined, most commonly as NYHA class II–IV (Figure 2).

The criteria defining eligibility for LV-only fCRTp were not standardized. This variability was apparent not just in comparative studies but also in those investigating true LV-only fusion pacing with dual-chamber RA/LV CRT devices. As detailed in Table 3, nearly all studies required the patients to be in sinus rhythm, though some allowed paroxysmal or persistent atrial fibrillation (AF). The definitions of preserved AV conduction varied: nine studies set a PR interval cutoff at ≤200 ms, while others accepted cutoffs of <240 or <250 ms (Table 3). Additionally, 18 studies specifically excluded patients with second- or third-degree AV block. Furthermore, bradycardia or pacing dependency served as exclusion criteria in 10 studies (Table 3). Functional assessment of AV conduction was employed in only a few studies, typically through VDD-mode screening or predefined run-in periods. These protocols were previously detailed in specific studies [13,14].

This heterogeneity in selection criteria reflects an ongoing effort to define which patients are most likely to benefit from LV-only fCRTp and to ensure consistent fusion over time. These findings raise an important clinical question: could a better patient selection refine mid- and long-term outcomes in LV-only fCRTp?

**Table 1:** Summary of trials and studies comparing LV-only fusion pacing with BiVp for CRT.

Scheme 2001.	Trial Name (if Applicable)	Study Design	Number of Patients	Inclusion Criteria	Exclusion Criteria for LV Only	FOI Method	FU	Results
**Touiza, 2001 [15]**	-	prospective, observational	total = 33 LV-only fCRTp = 18	ischemic and nonischemic etiology, QRS ≥ 140 ms, LBBB, SR	-	Echo	6 months	similar response; mortality: 7 deaths (3 in BiV, 4 in LV group); only 1 sudden death (BiV group)
**Etienne, 2001 [16]**	-	prospective, observational	total = 23 LV-only fCRTp = 13	ischemic and nonischemic etiology, NYHA class III–IV LVEF ≤ 40%, LBBB	patients without hemodynamic improvement during acute LV-based pacing (test response)	Echo	8 ± 3 months	similar response; no difference in response between patients in SR and AF
**Gasparini, 2006 [17]**	**BELIEVE**	parallel-group randomized controlled trial	total = 69 LV-only fCRTp = 36	ischemic and nonischemic etiology; NYHA class II-IV, LBBB, ICD indication	AF; pm dependency	Echo	12 months	similar response; no difference in adverse events; no AV block at FU
**Rao, 2007 [13]**	**DECREASE-HF**	parallel-group randomized controlled trial	total = 306 BiVp (vv = 0) vs. BiV (VVi optimized) vs. LV-only fCRT 1:1:1	ischemic and nonischemic etiology; NYHA class III-IV; ICD indication; 2 weeks of VDD mode before randomization	β-blocker therapy for ≤90 days	None	6 months	similar response; BiV (VV = 0) demonstrated a greater reduction in LVESD; no significant differences in adverse events
**Sirker, 2007 [18]**	**LOLA-ROSE**	crossover randomized controlled trial	total = 18	ischemic and nonischemic etiology; NYHA class III-IV; LBBB, LV mechanical dyssynchrony	-	Echo	2 × 8 weeks	similar response; no difference in adverse events
**Valzania, 2008 [19]**	-	parallel-group randomized controlled trial	total = 22	ischemic and nonischemic etiology, NYHA class III-IV, LBBB	AF; 2nd- or 3rd-degree AV block, severe renal dysfunction	Echo	3 months	similar response
**Boriani, 2010 [20]**	**B-LEFT HF**	parallel-group randomized controlled trial	total = 176 LV-only fCRT = 86	ischemic and nonischemic etiology; NYHA class III-IV; ICD indication	AF, 2nd- or 3rd-degree AV block, pacing for bradycardia	-	6 months	similar response; no difference in adverse events
**Sedlacek, 2010 [21]**	-	parallel-group randomized controlled trial	total = 40	nonischemic etiology; NYHA class III-IV	AF, 2nd- or 3rd-degree AV block, pacing for bradycardia	Echo	3 years	greater improvement in LVEF, LV remodeling in BiVp; higher CV mortality in LV-only fCRT, higher incidence of arrhythmic events, and a greater necessity for CRT-D upgrade in LV-only fCRT
**Thibault, 2011 [14]**	**GREATER-EARTH**	crossover randomized controlled trial	total = 121	ischemic and nonischemic etiology, 6 min walk test distance ≤ 400 m, ICD indication, PRi ≤ 250 ms; Ap/Vp < 5% in “run-in” test;	AF, 2nd- or 3rd-degree AV block, pacing for bradycardia	EKG	2 × 6 months	similar response; 17.1% of BiV non-responders improved with LV-only fCRTp
**Martin, 2012 [22]**	**ADAPTIV-CRT**	parallel-group randomized controlled trial	total = 522 aBiV/LV-only fCRTp = 219	ischemic and nonischemic etiology, NYHA class III-IV, ICD indication	AF, 2nd- and 3rd-degree AV block	aCRT	9.7 ± 3 months	significant improvement in clinical response in aCRT group; no significant difference in cardiac output, HF hospitalizations, or mortality rates between aCRT and BiV pacing (Echo-optimized)
**Birnie, 2013 [23]**	**ADAPTIV-CRT**	parallel-group randomized controlled trial	total = 478 aBiV/LV-only fCRTp = 142	ischemic and nonischemic etiology, ICD indication	AF	aCRT	1 year	LV-only fCRTp ≥ 50%: a greater improvement in NYHA class, LVEF, and LV remodeling; significant reduction in mortality and HF hospitalizations
**Burns, 2017 [24]**	**ADAPTIV-CRT**	parallel-group randomized controlled trial	total = 161 LV-only fCRTp = 70	ischemic and nonischemic etiology, LBBB, ICD indication	AF, AV block	aCRT	12 months	greater improvement in LVEF, global LV myocardial strain in septal and apical regions with LV-only pacing; 77% responders in the LV-only group; (66% responders in BiV group)
**Faghfourian, 2017 [25]**	-	crossover randomized controlled trial	total = 44	ischemic and nonischemic etiology, NYHA class II-IV, LBBB: 38 patients Non-LBBB: 7 patients, “preserved AV conduction”	AF, 3rd-degree AV block	-	2 × 3 months	similar response
**Gwag, 2019 [26]**	-	retrospective, observational	total = 155 convBiV = 129; aBiV = 11; aLV-only fCRT = 15	ischemic and nonischemic etiology, NYHA class II-IV, LBBB; CRT-D (94.2%)	AF	aCRT/Echo/EKG in convBiV	27.5 months	LVEF after 6 months: highest in aLV-only fCRT group; super-responders: 58.3% in aLV-only fCRT vs. 36.3% in aCRTBiV vs. convBiV 14.3%; no SCD or ICD upgrades in LV-only fCRTp group
**Hsu, 2019 [27]**	-	retrospective, observational	total = 37.450 aCRT = 11.566 convBiV = 25.884	ischemic and nonischemic etiology, NYHA class II-IV	AF	aCRT	15.5± 9.1 months	higher LV-only fCRTp % was associated with lower AF incidence: 1.3% AF (LV-only > 92%) vs. 22.4% AF (0–5% LV-only)
**Su, 2021 [28]**	-	parallel-group randomized controlled trial	total = 73 nLV-only fCRTp = 34	ischemic and nonischemic etiology, NYHA class II-III; LBBB, PRi ≤ 200 ms	AF, AV block, pacing for bradycardia	aCRT *	6 months	higher super-response rate in LV-only fCRTp group (68.4%) vs. BiVp group (36.4%); no significant differences in adverse outcomes
**Vatasescu, 2023 [29]**	-	retrospective, observational	total = 622 nLV-only fCRTp = 408	ischemic and nonischemic etiology; NYHA class II-IV; PRi < 250 ms; Wenckebach point during atrial pacing < 500 ms	AF	EKG	6 months	the super-responder rate was highest in the LV-only pacing (41.91%) compared with BiVp group (9.81%)

AF—atrial fibrillation; AV block—atrioventricular block; aCRT—adaptive cardiac resynchronization therapy; convBiV—conventional biventricular pacing (VVi = 0 ms); CRT-D—cardiac resynchronization therapy with defibrillator; fCRTp—cardiac resynchronization therapy fusion pacing; Echo—echocardiography; EKG—electrocardiogram; FOI—fusion-optimized interval; FU—follow-up; LV—left ventricle; LVEF—left ventricular ejection fraction; LVESD—left ventricular end-systolic diameter; LBBB—left bundle branch block; total—total number of patients in the study; NYHA—New York Heart Association (functional classification); pm dependency—pacemaker dependency; PRi—PR interval; SCD—sudden cardiac death; SR—sinus rhythm; VDD mode—pacing mode with atrial sensing and ventricular pacing; Vp—ventricular pacing; * intentional no RV capture pacing mode.

**Table 2:** Summary of studies with left univentricular pacing (without RV lead).

Study, Year	Trial Name (if Applicable)	Number of LV-Only Patients	Inclusion Criteria	Exclusion Criteria	Type of Device Used	FOI Method	FU	Outcomes
**Auricchio, 2003 [30]**	**PATH-CHF II trial**	86	ischemic and nonischemic etiology; NYHA class III-IV	AF, 2nd- or 3rd-degree AV block, indication for pacing or ICD therapy	RA/LV DDD device	Echo	1–3 months -pacing ON/1 month-pacing OFF	in patients with QRSd ≥ 150 ms, there is a significant increase in LVEF, improvement in NYHA class and quality of life, no AV block, and no need for upgrade to ICD
**Blanc, 2004 [31]**	**PATH-CHF II trial**	22	ischemic and nonischemic etiology, NYHA class III-IV, LBBB	AF, AV block, indication for pacing	RA/LV DDD device	Echo	12 months	NYHA class improved by 40%; LVEF improved by 22%; reverse remodeling (LVEDD reduced by 5%, LVESD reduced by 10%, MR area reduced by 40%; mortality: 23% (5 from HF, 2 SCD)
**Butter, 2006 [32]**	-	29	ischemic and nonischemic etiology, NYHA class III-IV; LBBB	AF, 2nd- or 3rd-degree AV block, ICD indication	RA/LV VDD device	EKG	12 months	LVEF increased from 23 ± 7% to 34 ± 9%; QRS duration shortened significantly from 167 ± 21 ms to 140 ± 17 ms; clinical improvement: NYHA class, no AV block, no need for upgrade to ICD
**Gopi, 2014 [33]**	-	5	nonischemic DCM, NYHA class III-IV, LBBB	AF, AV block, bradycardia, renal dysfunction (serum creatinine > 1.5 mg/dL)	RA/LV VDD device	EKG	6 months	QRS d shortened from 174 ± 17 ms to 128 ± 10.9 ms; LVEF improved from 25 ± 6% to 34 ± 6%; reverse remodeling (LVEDD decreased from 73.2 ± 12 mm to 65.8 ± 9.6 mm; LVESD reduced from 65 ± 12 mm to 54 ± 10 mm; no complications
**Zhao, 2017 [34]**	-	30	ischemic and nonischemic etiology, NYHA class II-IV, LBBB; “preserved AV conduction”	AV block	RA/RV/LV device with deactivated RV lead; RA/LV DDD device	EKG and Echo/RAAVD	7.86 ± 3.67 months	clinical improvement: NYHA class, 6MWT, peak VO_2_; LVEF improved by 9.7%(conv BiVp 6.8%); QRS d shortened from 167.5 ms to 139.2 ms in LV-only fCRTp; no significant difference in mortality
**Pu, 2017 [35]**	-	36	ischemic and nonischemic etiology, NYHA class II-IV, LBBB; PQ interval < 220 ms	AF, 2nd- or 3rd-degree AV block	RA/RV/LV device with deactivated RV lead; RA/LV DDD device	EKG and Echo/RAAVD	13 months	QRS d shortened from 182 ± 20 ms to 132 ± 9.8 ms; LVEF improved from 27% ± 6% to 41% ± 9%; reverse remodeling (MRA reduction from 4.3 ± 1.2 cm^2^ to 1.9 ± 1.1 cm^2^); no significant difference in mortality; device longevity comparison between LV-only fusion p and convBiV-CRT: 6.9 ± 0.3 years to 3.7 ± 0.2 years
**Cozma, 2018 [36]**	-	55	nonischemic etiology, NYHA class II-III, LBBB; PRi ≤ 250 ms	ischemic etiology, AF, 2nd- or 3rd-degree AV block/ history of syncope/ Wenckebach point < 500 ms; ICD indication	RA/LV DDD device	EKG during rest and during stress test	35 ± 18 months	significant LV reverse remodeling (LVEDV decreased from 243.2 ± 82 mL to 193.7 ± 81 mL; reduction in mitral regurgitation severity was noted in 38 patients (69%); LVEF improved from 27 ± 5.2% to 38 ± 7.9%; one patient developed 2nd degree AV block and upgrade to a triple-chamber CRT-p needed
**Goanta, 2022 [37]**	-	83	nonischemic etiology, NYHA class II-III, LBBB; PRi < 240 ms	ischemic etiology, AF, 2nd- or 3rd-degree AV block/ ICD indication; Wenckebach point < 500 ms; structural cardiomyopathies or channelopathies with a risk of SCD	RA/LV DDD device	EKG during rest and during stress test	6 months	SRs: 25/83 (31%) with ≥30% LVESV reduction and LVEF ≥ 45%; SR predictors: higher baseline LVEF, lower PASP, less severe MR; no SCD in SR group; no need for ICD upgrade
**Gurgu, 2024 [38]**	-	62	nonischemic etiology, NYHA class III-IV, LBBB; preserved AV conduction	ischemic etiology, AF, ICD indication	RA/LV DDD device	Echo	45 ± 19 months.	LVEF, NYHA class, significantly improved; improvement in diastolic dysfunction (64%); CRT-P upgrade for AV block: 4 pts (5.5%); no SCD, and no ICD upgrades were required
**Vacarescu, 2024 [39]**	-	73	nonischemic etiology, NYHA class II-III, LBBB; PRi < 240 ms	ischemic etiology, AF, 2nd- or 3rd-degree AV block/history of syncope; ICD indication; structural cardiomyopathies or channelopathies with a risk of SCD	RA/LV DDD device	EKG during rest and during stress test	6.4 years ± 27 months	LVEF improved from 27.9 ± 5.1% to 40.4 ± 8.5%; reduction in mitral regurgitation severity in 69%; LA responder = significant LA reverse remodeling; AV block and upgrade to a triple-chamber CRT-p needed in 4%; mortality: 7% (2 from HF/3 extracardiac causes)

AF—atrial fibrillation; AV block—atrioventricular block; DDD device—dual-chamber pacing device; Echo—echocardiography; EKG—electrocardiogram; FOI—fusion-optimized interval; FU—follow-up; HF—heart failure; ICD—implantable cardioverter-defibrillator; PRi—PR interval; LA—left atrium; LBBB—left bundle branch block; LV—left ventricle; LVEDD—left ventricular end-diastolic diameter; LVEDV—left ventricular end-diastolic volume; LVEF—left ventricular ejection fraction; LVESD—left ventricular end-systolic diameter; LVESV—left ventricular end-systolic volume; MR—mitral regurgitation; MRA—mitral regurgitation area; NYHA—New York Heart Association (functional classification); PASP—pulmonary artery systolic pressure; QRSd—QRS duration; RA—right atrium; RAAVD—right atrial-based AV delay optimization; RV—right ventricle; SCD—sudden cardiac death; SR—super-responder; VDD device—device with atrial sensing and ventricular pacing.

**Table 3:** Summary of specific selection criteria for LV-only fCRTp.

Specific Selection Criteria for LV Only								
Inclusion Criteria					Exclusion Criteria			
Criteria			Number of Studies	References	Criteria		Number of Studies	References
**sinus rhythm**			**26/27**	[13,14,15,17,18,19,20,21,22,23,24,25,26,27,28,29,30,31,32,33,34,35,36,37,38,39];	**atrial fibrillation**	**paroxysmal**	**9/27**	[17,25,26,31,33,35,37,38,39]
						**persistent**	**9/27**	[14,17,20,21,25,26,28,33,35]
						**permanent**	**24/27**	[14,17,18,19,20,21,22,23,24,25,26,27,28,29,30,31,32,33,34,35,36,38,39]
**Normal AV conduction**	**PR interval** **functional tests**	**PRi ≤ 200 ms**	**9/27**	[22,23,24,26,27,28,30,32,34]	**chronotropic insufficiency/pacing for bradycardia/pacemaker dependency**		**10/27**	[13,14,17,19,20,21,22,28,31,33]
		**PRi < 240 ms**	**2/27**	[37,39]	**heart rate at baseline > 100 bpm**		**5/27**	[22,23,24,26,27]
		**PRi < 250 ms**	**3/27**	[14,29,36]	**2nd- or 3rd-degree AV block**		**18/27**	[14,19,20,21,22,24,25,28,30,31,32,33,34,35,36,37,38,39]
		**PQi < 220 ms**	**1/27**	[35]	**others**			
		*Wenckebach point during atrial pacing > 500 ms*	**3/27**	[29,36,37]		*structural cardiomyopathies or channelopathies with a risk of SCD*	**2/27**	[37,39]
		*2–8 weeks of “run-in” test before randomization*	**1/27**	[14]		*LV dyssynchrony on Echo*	**1/27**	[18]
		*improvement during acute LV-based pacing (test response) before CRT*	**1/27**	[16]		*ICD indication in secondary prevention (for RA/LV devices)*	**6/10**	[30,32,36,37,38,39]
		*2 weeks of VDD testing mode before randomization*	**1/27**	[13]		*Severe renal dysfunction*	**3/27**	[19,33,34]

AV block—atrioventricular block; Echo—echocardiography; ICD—implantable cardioverter-defibrillator; LV—left ventricle; PQi/PRi—PQ/PR interval; RA—right atrium; RA/LV device—dual-chamber device with right atrium and left ventricle leads; SCD—sudden cardiac death; VDD—pacing mode with atrial sensing and ventricular pacing.

Risk of bias and quality assessment

**Figure A1:**
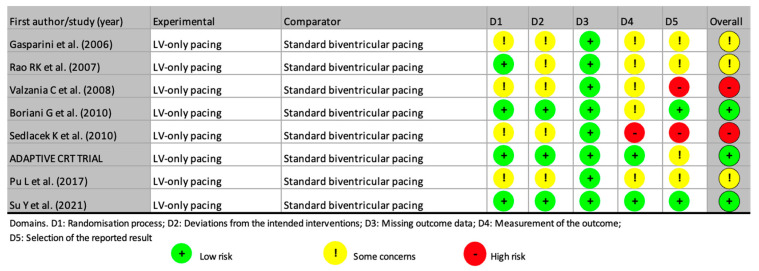
Risk of Bias 2.0 assessment for parallel-group randomized controlled trials [13,17,19,20,21,22,23,24,28,35].

**Figure A2:**
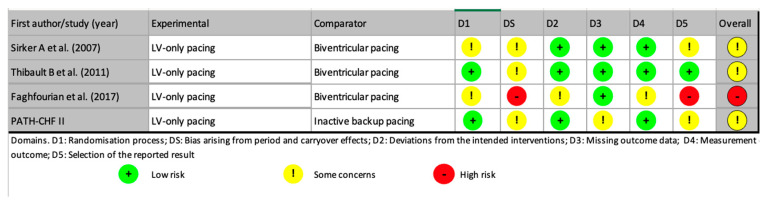
Risk of Bias 2.0 assessment for crossover randomized controlled trials [14,18,25,30,32].

Risk of bias was assessed using the Cochrane RoB 2.0 tool for randomized trials and the MINORS criteria for non-randomized studies. Among parallel-group RCTs (Appendix A, Figure A1; Supplementary Materials, S2), the overall risk of bias was low in three studies. Other studies presented some bias concerns, either due to a lack of predefined, open-source study protocols or because of some subjectivity regarding outcome measurement and selective reporting. Two trials had a high risk due to selective outcome reporting. For crossover trials (Appendix A, Figure A2; Supplementary Materials, S3), three of four studies had a moderate risk, and the other had a high risk of bias.

**Figure A3:**
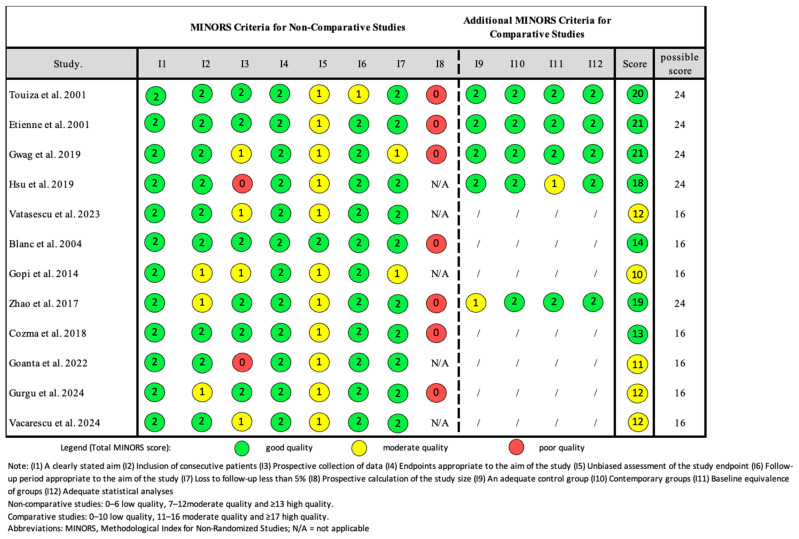
MINORS quality assessment for non-randomized studies [15,16,26,27,29,31,33,34,36,37,38,39].

Non-randomized studies (Appendix A, Figure A3) were generally of good quality. Based on MINORS scoring, 7 of 13 studies reached the threshold for high quality, while the rest had a moderate quality, according to the assessment protocol. Common limitations included a lack of prospective size calculation, follow-up issues, or some bias regarding the assessment of the study endpoints.

### 3.1. Studies Reporting Similar Outcomes Between LV-Only Fusion Pacing and BiVp

**Figure 3:**
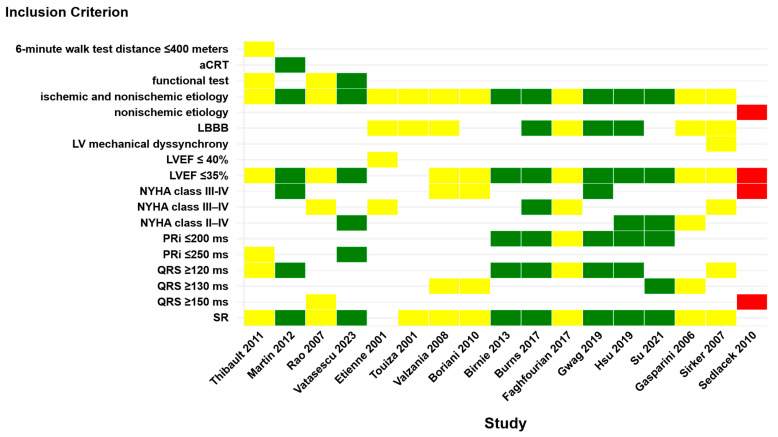
Comparative heatmap summarizing the inclusion criteria and clinical outcomes of studies evaluating LV-only fCRTp versus BiVp. Each column represents a study, while each row denotes a specific inclusion criterion. Color coding reflects the clinical outcome of LV-only fCRTp compared to BiVp: yellow—similar outcomes between LV-only fCRTp and BiVp; green—superior outcome with LV-only fCRTp; red—inferior outcome with LV-only fCRTp [13,14,15,16,17,18,19,20,21,22,23,24,25,26,27,28,29].

Nine comparative studies reported similar clinical outcomes between LV-only fCRTp and conventional BiVp [13,14,15,16,17,18,19,20,25] (Table 1, Figure 3). These studies included patients with both ischemic and nonischemic cardiomyopathy (Table 1, Figure 3). The most common baseline characteristics found in studies were sinus rhythm, LBBB morphology, QRS duration ≥ 120 ms (typically between 130 and 150 ms), and NYHA functional class II to IV. These studies, conducted between 2001 and 2017, include a mix of randomized controlled trials, with either crossover or parallel-group design, and prospective or retrospective observational studies. These were among the earliest attempts to report LV-only fCRTp as a potentially equivalent alternative to BiVp.

**Figure 4:**
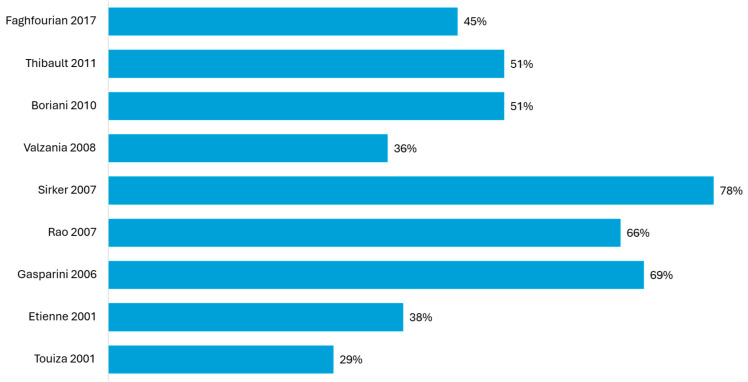
Proportion of ischemic patients in LV-only fCRT group from studies that reported similar outcomes with BiVp [13,14,15,16,17,18,19,20,25].

The proportion of ischemic cardiomyopathy in LV-only groups varied widely, ranging from 29% to 78% (Figure 4). The earliest observational studies conducted by Touiza and Etienne (2001) [15,16] included a relatively low percentage of ischemic patients (29% and 38%, respectively), while the latter studies included more balanced populations (Figure 4).

Four studies reported CRT-D use for primary prevention of SCD in patients with ischemic cardiomyopathy [13,14,17,20] (Table 1). Five studies either used CRT-P systems or did not specify defibrillator use [15,16,18,19,25] (Table 1).

Touiza [15] and Etienne [16] described about 20% and 30% mortality over 6–8 months, though these were early observational studies without standardized selection protocols or ICD backup. In contrast, more recent trials such as BELIEVE [17], B-LEFT HF [20], GREATER-EARTH [14], and DECREASE-HF [13] showed no significant differences in all-cause mortality or adverse event rates between LV-only fCRT and BiVp arms at 6 to 12 months. No deaths or arrhythmic events were reported in Valzania [19], LOLA-ROSE [18], or Faghfourian [25]. Among all these studies, none identified a higher incidence of SCD or ventricular arrhythmias in the LV-only fCRTp group compared to BiVp (Table 1).

Analyzing the nine studies that reported comparable outcomes between LV-only fCRTp and BiVp reveals substantial inconsistency in how preserved AV conduction was defined and applied. Early observational studies, such as those conducted by Touiza [15] and Etienne [16], lacked formal AV conduction criteria and relied primarily on sinus rhythm to select patients for LV-only fCRTp. Furthermore, in Etienne’s cohort, 9 out of 23 patients were in AF, and some of them still received LV-only CRT. The only exclusion criterion reported in that study was the absence of an acute hemodynamic response to pre-implantation LV-based pacing [16]. In contrast, subsequent studies applied more structured and physiology-guided selection protocols [13,14,17,18,19,20,25]. Sinus rhythm was a standard requirement, and most studies excluded patients with permanent AF (Table 4). Atrial arrhythmias such as paroxysmal and persistent AF were variably handled, accepted in some studies [13,18,19], but explicitly excluded in others [17,25] (Table 4).

Only a subset of studies provided quantitative thresholds for preserved AV conduction (Table 4). GREATER-EARTH [14] set a PR interval cutoff of <250 ms and excluded patients with second- or third-degree AV block, a criterion also applied in Valzania [19], B-LEFT HF [20], and Faghfourian [25]. Functional screening tests, such as a two-week VDD-mode run-in phase in DECREASE-HF [13] and prolonged AV delay testing in GREATER-EARTH [14], were used to evaluate real-life intrinsic AV conduction and stability before randomization. Several studies introduced additional exclusion filters to ensure long-term constant fusion. Patients with pacing dependency or bradycardia indications were excluded in BELIEVE [17], DECREASE-HF [13], Valzania [19], B-LEFT HF [20], and GREATER-EARTH [14]. Mechanical dyssynchrony assessed by echocardiography was required only in one trial [18], and DECREASE-HF [13] underscored the importance of stable beta-blocker therapy prior to randomization by excluding patients who had initiated treatment within the preceding 90 days.

### 3.2. Studies Reporting Inferior Outcomes with LV-Only Fusion Pacing

Only one study reported inferior outcomes with LV-only fCRTp compared to BiVp. Sedláček et al. conducted a single-center, parallel-group, randomized trial including 40 patients with nonischemic cardiomyopathy and conventional CRT indication (NYHA class III–IV, LVEF < 35%, QRS ≥ 150 ms, LVEDD ≥ 55 mm) (Figure 3). Patients with AF, pacing indications for bradyarrhythmia, or non-LBBB morphology were excluded. Patients were randomized to either BiVp or isolated LV-only fCRTp. Functional assessments and echocardiography were performed at baseline and after 3, 6, and 12 months. A cross-sectional analysis of major adverse events was conducted at a median follow-up of 3 years. The study cohort represented a clinically advanced HF population, with a mean LVEF of ~21%, QRS duration > 190 ms, and NYHA class >III. At 12 months, patients in the BiVp group showed significantly greater improvements in LVEF (+12.5% vs. +5.1%, *p* = 0.01) and LVEDD reduction (−8.7 mm vs. −5.1 mm, *p* = 0.05). After 3 years, cardiovascular mortality occurred only in the LV-only group (three deaths), while no cardiovascular deaths were reported in the BiVp group. Additionally, five patients in the LV-only fCRTp group required CRT-D upgrades, compared to one in the BiVp group [21].

### 3.3. Studies Reporting Superior Outcomes with LV-Only Fusion Pacing

A subset of seven studies have shown that LV-only fCRTp may offer superior outcomes compared to traditional BiVp in selected patients, particularly in terms of LVEF improvement, reverse remodeling, higher responder rates, and clinical outcomes [22,23,24,26,27,28,29] (Table 1, Figure 3).

**Figure 5:**
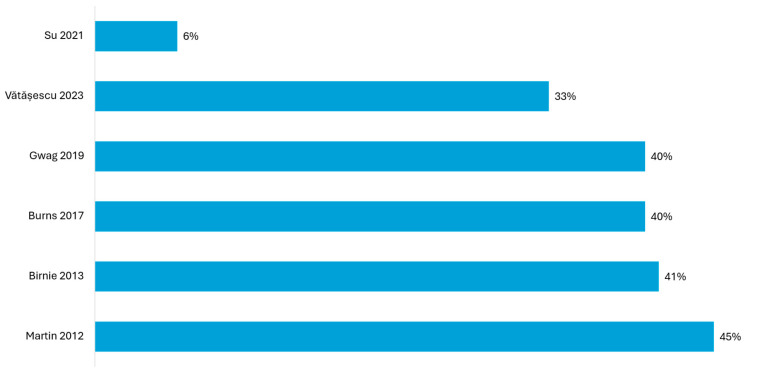
Proportion of ischemic patients in studies reporting superior outcomes with LV-only fCRTp [22,23,24,26,28,29].

The proportion of ischemic cardiomyopathy ranged from 5.9% to 45%, with most studies enrolling predominantly nonischemic patients (Figure 5). Notably, this ischemic burden was consistently lower compared to that reported in the studies showing similar outcomes between LV-only fCRTp and BiVp (Figure 4).

All seven studies included patients with HF who met standard CRT criteria: LVEF ≤ 35%, symptomatic status corresponding to NYHA class II–III, typical LBBB morphology, and QRS duration ≥ 120 ms (most often exceeding 150 ms on average). All patients were in sinus rhythm, and permanent AF was an exclusion criterion in all studies (Table 5).

In most studies reporting superior outcomes, LV-only fCRTp was delivered using the adaptive CRT (aCRT) algorithm. Among the seven studies, only the trial conducted by Vătășescu did not use aCRT. In this study, patient selection and fusion optimization were based on a physiology-driven strategy. Inclusion criteria required a PR interval < 250 ms and a Wenckebach point > 500 ms, assessed via atrial pacing to ensure stable AV conduction. AV delays were manually optimized using surface ECGs at rest and during exercise testing. This individualized, physiology-guided method resulted in a super-responder rate of 41.9% in the LV-only fCRTp group, significantly higher than the 9.8% observed in the BiVp group (*p* < 0.0001) [29].

In large real-world data, Hsu et al. found that a higher percentage of aLV-only fCRTp was associated with a significantly lower incidence of AF. Furthermore, a PR interval ≤ 200 ms independently predicted this benefit, suggesting a key role for intrinsic conduction in achieving favorable outcomes with LV-only fCRTp [27].

Several trials consistently demonstrated that the clinical benefits of LV-only fCRTp were closely tied to the proportion of effective fusion achieved (Table 6). In Martin et al.’s study, superior outcomes were observed in the subgroup with preserved AV conduction and an LV-only fCRTp percentage around 64%, suggesting that the benefits of adaptive CRT depend on reaching a sufficient fusion pacing percentage (fCRTp%) [22]. Birnie et al. further confirmed this dose-dependent response relationship, showing that patients with ≥50% LV-only fCRTp experienced significantly fewer HF hospitalizations and deaths, with fCRTp% averaging 68–73% during follow-up [23]. Similar findings were reported by Burns, where patients with preserved AV conduction showed greater improvements in LVEF and myocardial strain with LV-only fCRTp compared to BiVp [24].

In the study conducted by Gwag, patients with advanced HF undergoing CRT were randomized into three groups based on optimization strategy: non-adaptive BiVp (echo/EKG optimized BiVp), adaptive BiVp, and adaptive LV-only fCRTp. Among patients in the adaptive LV group (n = 15), the median LV-only fCRTp% was 97.7%. The super-responder rate in this group was 58.3%, compared to 36.3% in the non-adaptive BiVp group and 14.3% in the adaptive BiVp group. Super-response was defined as a clinical responder (survival at 6 months with NYHA class improvement) with either a ≥30% relative reduction in LVESV or post-CRT LVEF ≥ 45% [25]. Finally, Su et al. extended these findings in a recent RCT comparing adaptive LV-only fCRTp using the AdaptivCRT™ algorithm (n = 34) with echocardiography-optimized BiVp (n = 29). Importantly, this study implemented intentional non-capture of the RV by programming the output to sub-threshold levels, ensuring true LV-only fCRTp. In the subgroup with high-percentage adaptive LV-only fCRTp (n = 25), the super-responder rate was 68.4%, significantly higher than in the BiVp group (36.4%, *p* = 0.04). Super-response in this study was defined by a composite of at least a two-fold increase in LVEF or final LVEF > 45%, LVESV reduction > 15%, and ≥1 NYHA class improvement [28].

### 3.4. True LV-Only Fusion CRT Using RA/LV Dual-Chamber Devices

A total of 481 patients across 10 studies received dual-chamber CRT devices programmed in RA/LV DDD or VDD mode, omitting the RV lead, to achieve true LV-only fCRTp [30,31,32,33,34,35,36,37,38,39] (Table 2). These groups included three randomized controlled trials [30,34,35] and seven prospective observational studies [31,32,33,36,37,38,39]. Fusion optimization strategies varied, including echocardiography-guided AV delay programming, surface ECG algorithms at rest and during exercise testing, and rate-adaptive AV delay protocols.

All patients who received RA/LV dual-chamber CRT devices had HF with a LVEF < 35%, LBBB, and were on optimized GDMT, with the majority presenting in NYHA functional class III or IV. Approximately half of the included studies enrolled patients with an ischemic etiology of cardiomyopathy. Among the ten included studies, three adopted a strict intrinsic PR interval cutoff ≤ 200 ms [30,32,34]. One study accepted values up to 220 ms [35], two allowed a maximum of 240 ms [37,39], and only one study proposed a threshold below 250 ms [36] (Table 3). In addition, three other studies did not define a specific PR interval value at baseline but included only patients with “preserved AV conduction”, implying physiologic PR intervals likely under 200 ms [31,33,38] (Table 2). All studies excluded patients with second- or third-degree AV block and permanent AF, as these conditions prevent consistent fusion pacing. Furthermore, the need for an ICD for secondary SCD prevention was a common exclusion criterion. Moreover, three studies [37,38,39] excluded patients with inherited structural cardiomyopathies or channelopathies associated with an increased risk of SCD (Table 3). To ensure stable AV conduction, two studies required a Wenckebach point greater than 500 ms to exclude patients with concealed or rate-dependent AV block [36,37].

Table 7 summarizes the mid- and long-term clinical benefits and complications reported across the 10 studies investigating true LV-only fCRTp using RA/LV dual-chamber devices. RA/LV dual-chamber fusion pacing consistently corrected electrical dyssynchrony, with QRS narrowing reported across multiple studies. In Gopi et al. [33], QRS duration was reduced from a mean value of 174 ± 17 ms to 128 ± 10.9 ms, and in the randomized trial conducted by Pu et al., from 182 ± 20 ms to 132 ± 9.8 ms [35]. LVEF improvement was observed in all included studies. Notably, Goanta reported 31% super-responders, defined as patients with ≥30% LVESV reduction and LVEF ≥ 45%, at the 6-month follow-up assessment [37]. This favorable systolic response was confirmed by Cozma et al., reporting an LVEF increase from 27 ± 5.2% to 38 ± 7.9% over 35 ± 18 months [36].

LV reverse remodeling emerged as a consistent marker of CRT response, with several studies specifically quantifying reductions in LV end-systolic and end-diastolic volumes. Notably, in the study conducted by Cozma, LVEDV decreased from 243.2 ± 82 mL to 193.7 ± 81 mL [36]. At the same time, Gopi reported a reduction in LVESD from 65 ± 12 mm to 54 ± 10 mm, indicating significant systolic remodeling [33].

Mitral regurgitation was directly assessed as a response parameter in six studies [31,33,35,36,37,38], with consistent improvement after LV-only fCRTp (Table 7). Pu et al. showed a marked reduction in mitral regurgitation area from 4.3 ± 1.2 cm^2^ to 1.9 ± 1.1 cm^2^ [35]. Furthermore, Blanc et al. documented a 40% reduction in MR area over 12 months [31], and Vacarescu et al. reported MR severity reduction in 69% of patients [39].

Beyond systolic parameters, diastolic dyssynchrony and function were explored in detail by Gurgu, who introduced the novel echocardiographic markers E″T and A″T to quantify diastolic dyssynchrony. Their study showed that correcting E″T from 90 ± 20 ms to 25 ± 10 ms was associated with significant LV reverse remodeling and improved filling pressures (E/E′ ratio) [38]. Moreover, LA (left atrium) remodeling was also reported in four studies as a secondary benefit of improved LV synchrony [31,36,38,39] (Table 2). For instance, Vacarescu et al. defined LA responders as those with significant volume reduction and linked this with decreased supraventricular arrhythmic burden [39]. Similarly, Gurgu et al. reported improved diastolic parameters and LA volume alongside lower rehospitalization rates and stable sinus rhythm in most responders [38].

Two studies underscored the economic advantage of using dual-chamber RA/LV devices for CRT. Gopi et al. showed that in patients who could not afford standard CRT, LV-only pacing with a VDD pacemaker reduced costs by over 50% while delivering clinical benefit [33]. Similarly, Pu et al. demonstrated substantial cost savings by eliminating the RV lead and reported superior device longevity (6.9 ± 0.3 vs. 3.7 ± 0.2 years, *p* < 0.001) [35].

The studies including ischemic cardiomyopathy patients consistently reported high complication and mortality burdens. In the observational study conducted by Blanc, 32% of patients had ischemic cardiomyopathy. Despite notable clinical and echocardiographic improvements, the 12-month mortality reached 23% (including two sudden deaths without ICD backup) [31]. Similarly, in the Butter et al. study, with 34% ischemic patients, mortality reached 17%, again without ICD use [32].

Zhao and Pu conducted a comparison of LV-only pacing with optimized AV delay versus standard BiVp. They enrolled lower proportions of ischemic patients (13.9% and 16.7%, respectively). Similar efficacy to BiVp was found in terms of functional class, hemodynamics, and LV reverse remodeling, with low all-cause mortality (3–4 deaths per group over 13 months) [34,35].

The PATH-CHF II crossover trial conducted by Auricchio included 38% patients with coronary artery disease and demonstrated that LV-only fCRTp significantly improved peak VO_2_, 6 min walk distance, and quality of life, particularly in patients with QRS ≥ 150 ms. However, all five deaths during the crossover phase occurred in patients without ICD backup, four of which were sudden cardiac deaths [30].

The occurrence of AV block and the subsequent need for device upgrade represent essential safety considerations in LV-only fCRTp. Across the included studies, the incidence of AV block was low. However, three studies reported late-onset AV conduction deterioration requiring upgrade to conventional triple-chamber CRT systems. In the study conducted by Cozma, one patient (1.8%) developed second-degree AV block during a mean follow-up of 35 ± 18 months [36]. Similarly, Gurgu reported four patients (6.5%) required BiVp upgrade due to progressive AV block over a 45 ± 19-month follow-up [38]. Vacarescu demonstrated an AV block occurrence in 4% of patients during a 6.4 ± 2.3-year follow-up [39]. Finally, none of the studies reported a need for ICD or CRT-D upgrade for secondary prevention during follow-up.

## 4. Discussion

The most recent systematic review focusing on LV-only fCRTp was published by Burri et al. in 2017 on behalf of the EHRA Education Committee [7]. It included six randomized trials comparing LV-only fCRTp with BiVp and concluded that clinical outcomes were broadly similar in selected populations [7]. However, the present systematic review specifically aims to refine LV-only fCRTp by identifying the most suitable candidates, an area that has not been systematically addressed until now. By including 27 studies published between 2001 and 2024, covering all types of study designs and various fusion pacing strategies, our analysis provides the most comprehensive synthesis of selection criteria that may influence mid- and long-term outcomes in LV-only fCRTp (Table 1 and Table 2, Figure 3). Furthermore, it offers a broader perspective on the clinical performance by including 10 studies using RA/LV dual-chamber devices without an RV lead, a novel and underreported subgroup in the literature, to our knowledge (Table 2).

A central finding across this body of evidence is the marked heterogeneity in patient selection strategies for LV-only fCRTp, which appears closely linked to the variability in clinical outcomes, ranging from less favorable, non-inferior, and even to superior results when compared to BiVp (Figure 3). Considering that all studies applied the same CRT indications, defined by guideline recommendations that were in use at the time of each study, these were considered standard criteria for resynchronization. Specifically, the most prevalent are symptomatic HF, LVEF ≤ 35%, prolonged QRS duration, and LBBB morphology. Earlier studies largely followed the 2005 ACC/AHA [40] and 2007 ESC guidelines [41], which broadly defined CRT eligibility based on reduced LVEF and wide QRS duration, often ≥120–130 ms, with less rigorous QRS morphology requirements. In contrast, studies conducted after 2013 reflected the ESC 2013 [42] and 2016 guidelines, which emphasized the predictive value of typical LBBB and QRS duration ≥ 150 ms, particularly in nonischemic HF [43].

Although standard, guideline-based CRT criteria were used to qualify patients for resynchronization, some supplementary variables aimed to refine a narrower subpopulation that would identify eligible patients for LV-only fCRTp. These terms, “standard” and “specific”, were arbitrarily coined in this review to differentiate evidence-based resynchronization criteria from concepts that emerged from the pooled analysis of inclusion criteria used in LV-only studies.

Variations in selection criteria involved the definition of preserved AV conduction, etiology-based decisions for ICD implantation, and the management of supraventricular arrhythmias as a selection filter for maintaining effective fusion. These findings underscore the need to examine the main determinants of patient response to LV-only fCRT in greater depth.

An important consideration when interpreting the findings of this review is the amount of bias associated with the included studies. Overall, randomized trials exhibited low to moderate risk of bias. Most of them employed appropriate randomization methods and outcome reporting, though some lacked blinding or had limited power. The bias concern in the crossover studies was primarily due to carryover effects, particularly for an intervention such as CRT, where the washout period may have been insufficient to counter any residual impact. These suggest that while randomized studies were generally well-conducted, selective reporting and intervention deviations were recurrent issues. Non-randomized studies showed variable methodological rigor and require cautious interpretation of their findings. This variation in study quality must be considered when interpreting outcome heterogeneity across studies, and it further reinforces the need for high-quality, adequately powered trials to refine patient selection for LV-only fCRTp.

### 4.1. Variability in Defining Preserved AV Conduction

While AV conduction integrity is crucial for the success of LV-only fCRTp, there is currently no universally accepted definition of preserved AV conduction. Different studies apply varying thresholds. The aCRT algorithm defines normal AV conduction as a PR interval ≤ 200 ms if the patient is in sinus rhythm or ≤250 ms during atrial pacing. These thresholds are based on intracardiac electrograms, specifically the time from right atrial sensing to right ventricular sensing. When AV conduction is within these limits, the algorithm delivers synchronized LV-only pacing to preserve intrinsic RV activation and promote fusion. If the PR interval exceeds the cutoff, the device switches to BiVp to maintain effective ventricular synchrony. This dynamic strategy tailors real-time therapy to maximize hemodynamic benefit and reduce unnecessary RV pacing [23]. The rationale for this 200 ms cutoff comes from prior studies such as Kurzidim et al., who showed that patients with a PR interval ≤ 200 ms demonstrated greater improvement in contractility during LV pacing compared to those with longer intervals [44]. Nonetheless, all studies included in this review that utilized the aCRT algorithm were associated with superior outcomes for LV-only fCRTp compared to BiVp, with clinical benefits appearing to correlate directly with higher percentages of effective fusion pacing (Table 6).

Achieving a high rate of LV-only pacing using a PR interval cutoff of 200 ms may be challenging in real-world practice due to intrinsic AV conduction variability [45]. Effective fusion pacing has also been demonstrated in patients with longer PR intervals, including those exceeding 220–250 ms [14,29,35,36,37,39]. In a study conducted by Vacarescu et al., patients with first-degree AV block (PR interval > 200 ms) demonstrated greater stability of LV-only fCRTp during exercise than those with normal PR intervals. Only 21% of patients with long PRi lost LV capture during exercise, versus 39% in the normal PR group. Additionally, patients with longer PR required significantly less frequent AV delay reprogramming and fewer pharmacologic adjustments, indicating a more stable fusion window at higher heart rates [46]. These findings support that a modestly prolonged PR interval facilitates more constant and durable fusion pacing, even under stress conditions [47]. Therefore, the effectiveness of LV-only fCRTp in patients with PR > 200 ms depends less on a fixed baseline PR interval and more on individualized AV delay optimization and conduction stability over time.

Experimental studies have long demonstrated that AV conduction times vary significantly with changes in heart rate [48,49]. In patients with chronotropic incompetence, AV conduction may appear preserved at rest but becomes increasingly unstable at higher heart rates [50]. This phenomenon suggests a form of functional AV nodal incompetence, where conduction delays progressively with increasing atrial rates, as demonstrated in the RAVE study [51]. Such dynamic variability limits the predictability and reliability of maintaining consistent intrinsic right ventricular activation, which is critical for effective LV-only fCRTp. Therefore, relying exclusively on the resting PR interval to define preserved AV conduction may be insufficient. This limitation underscores the value of a more comprehensive, functional assessment of AV conduction. For example, in three observational studies, preserved AV conduction was defined not only by a resting PR interval but also by a Wenckebach point > 500 ms during atrial pacing [29,36,37]. This criterion served as a surrogate marker of long-term conduction stability, ensuring that intrinsic AV conduction would remain consistent across a range of heart rates and physiologic conditions [52,53]. Such an approach provides a more robust selection strategy for identifying candidates suitable for long-term LV-only fCRTp, beyond static surface ECG measurements. Similarly, trials like DECREASE-HF [13] and GREATER-EARTH [14] introduced real-life functional testing through “run-in periods” using prolonged AV delays, allowing intrinsic conduction to be monitored under ambulatory conditions. During these phases, the CRT device was temporarily reprogrammed to allow intrinsic conduction to dominate, and patients with low levels of ventricular pacing (Ap/Vp < 5%) were considered to have stable AV conduction. This approach served as a method for randomizing CRT candidates to LV-only or BiVp by monitoring AV conduction stability over time in real-life conditions, following titration of beta-blocker therapy.

### 4.2. Ischemic vs. Nonischemic Cardiomyopathy: Implications for LV-Only Fusion CRT

The underlying etiology of cardiomyopathy plays a pivotal role in the response to CRT. It has been consistently demonstrated that patients with nonischemic cardiomyopathy experience greater benefits from CRT, including more pronounced reverse remodeling and improved clinical outcomes. This concept, first described in 2003 [54], was subsequently reinforced by subgroup analyses of several major randomized controlled trials, such as MIRACLE [55], CARE-HF [56], REVERSE [57], and MADIT-CRT [58]. In contrast, in ischemic cardiomyopathy, the frequent presence of transmural or subendocardial scar tissue significantly impairs the response to CRT. Unlike nonischemic patients, where mechanical dyssynchrony primarily results from conduction delay, ischemic patients often present non-viable myocardial regions that cannot be recruited into effective contraction. This structural substrate not only limits the extent of reverse remodeling and reduces improvements in ejection fraction but also creates a vulnerable electrophysiological substrate, predisposing to ventricular arrhythmias [59].

In this analysis, ischemic burden emerged as a critical factor influencing the comparative outcomes between LV-only fCRTp and BiVp. The group of studies that reported similar outcomes between LV-only fCRTp and BiVp typically included moderate to high proportions of ischemic patients in the LV-only group, often ranging from 40% to over 75% (Figure 4). This contrasts with trials reporting superior outcomes with LV-only fCRTp, where nonischemic cardiomyopathy was predominant, and the ischemic burden was consistently below 45% (Figure 5). On the other hand, the study conducted by Sedláček is notable for being the only randomized trial to report inferior outcomes with LV-only fCRTp compared to BiVp, despite focusing exclusively on nonischemic patients. Over a median follow-up of three years, the LV-only group experienced higher cardiovascular mortality and a greater need for CRT-D upgrades. Notably, all cardiovascular deaths, including sudden cardiac death and deaths from heart failure progression, occurred in the LV-only group [21]. However, this study was characterized by a high risk of bias, primarily due to its small sample size, lack of blinding, and potential for selection bias (Appendix A, Figure A1). Furthermore, the study population consisted of patients with advanced heart failure (NYHA class III–IV), markedly reduced LVEF (~27%), and very wide QRS durations (~190 ms). This high-risk profile may have rendered these patients more susceptible to adverse outcomes regardless of pacing modality [60].

The current guideline-based indication for ICD implantation with the purpose of primary SCD prevention in patients with heart failure continues to rely only on a reduced LVEF (≤35%) [61]. However, risk stratification based exclusively on LVEF has limited specificity and sensitivity in predicting SCD. Notably, data from the Maastricht registry indicate that severe LV dysfunction was absent in most patients who developed SCD, underscoring the inadequacy of LVEF alone as a surrogate for arrhythmic risk [62]. While the benefit of ICD therapy in ischemic cardiomyopathy is well established, identifying nonischemic CRT recipients who may truly benefit from ICD backup remains a clinical challenge [63,64]. Data from the DANISH trial highlight this issue. Only 11.5% of ICD recipients received appropriate therapy for ventricular arrhythmias over a median follow-up of 68 months [65]. Importantly, the survival benefit was limited to patients ≤ 70 years, likely due to a higher competing risk of non-arrhythmic death in older patients [65].

Notably, the absence of an RV lead in RA/LV CRT devices has not been associated with adverse arrhythmic outcomes in studies enrolling predominantly nonischemic patients (Table 7). Across these cohorts, no CRT-D upgrades or sudden deaths were observed during follow-up, suggesting that with appropriate patient selection, devices without ICD backup may be safely considered [33,36,37,38,39]. Nonetheless, not all nonischemic patients carry the same arrhythmic risk, highlighting the necessity of refined and individualized risk stratification approaches [66,67]. This can be achieved using advanced imaging and genetic profiling. Cardiac magnetic resonance imaging (MRI) using late gadolinium enhancement (LGE) can identify mid-wall or subepicardial fibrosis. Values exceeding 13.7% of LV mass have been previously correlated with higher arrhythmic risk and diminished responsiveness to CRT [59,68]. Similarly, certain genetic mutations (e.g., LMNA, DSP, FLNC) may indicate a higher arrhythmic vulnerability [37,39].

Including these advanced tools in clinical decision-making may significantly enhance the selection between CRT-D and CRT-P, enabling a more personalized and risk-adapted therapy. In the context of LV-only fCRTp, the main concern is not necessarily AV block incidence but rather the absence of a defibrillator backup lead. Safety considerations were raised by some experts for patients with a potential risk for developing ventricular arrhythmias. Regarding the risk for developing AV block, Su et al. reported on six patients intentionally programmed for LV-only fCRTp by setting the RV lead output below the capture threshold. These patients experienced exclusive LV pacing without fusion, likely due to AV block development during follow-up (PRi > 200 ms). Despite the absence of fusion, they demonstrated clinical and echocardiographic outcomes comparable to those in the BiVp group, with no reported morbidity or mortality [28]. Furthermore, in studies evaluating the mid- and long-term outcomes of LV-only fCRTp using RA/LV dual-chamber devices, the incidence of AV block was found to be low (Table 7), and this did not increase morbi-mortality.

These findings highlight the critical role of accurate arrhythmic risk stratification in guiding optimal device selection. Nonischemic patients with preserved AV conduction and no evidence of myocardial fibrosis on cardiac MRI may be eligible for RA/LV dual-chamber fCRTp. In this carefully selected population, such an approach represents a more physiological, cost-effective, and potentially safe alternative to conventional BiVp systems [33,35].

### 4.3. Managing Supraventricular Arrhythmias to Preserve Constant Fusion Pacing

Effective LV-only fCRTp relies on stable intrinsic AV conduction to achieve fusion between paced LV activation and intrinsic RV activation. Supraventricular arrhythmias, particularly AF, can disrupt this synchrony by introducing unstable atrial activity and the loss of consistent AV conduction, compromising the fusion [69]. This explains why almost all studies in this review required sinus rhythm, and permanent AF was a universal exclusion criterion (Table 3). However, paroxysmal and persistent AF were managed variably. While some studies excluded both subtypes of AF, others allowed the inclusion of patients with paroxysmal and/or persistent AF (Table 3).

Therefore, data on the incidence of AF during LV-only fCRTp remain limited, with relatively few studies addressing this issue. Two comparative studies assessing aCRT versus conventional BiVp consistently demonstrated a lower incidence of AF in the aCRT arms (Table 1). In a large retrospective cohort study including over 37,000 CRT patients, Hsu et al. identified a strong negative correlation between the percentage of LV-only fCRTp and the incidence of AF. Patients were stratified by quartiles based on their LV-only fCRTp%, highlighting a clear downward trend in AF incidence over the two-year follow-up: from 22.4% in those with 0–5% LV-only fCRTp% to just 1.3% in patients with >92% LV-only fCRTp% [27]. These findings were further supported by prospective evidence from a randomized controlled trial conducted by Birnie, in which the aCRT group demonstrated a significantly lower incidence of sustained AF episodes compared to the conventional BiVp group (8.7% vs. 16.2%) over an average follow-up of 20 months [23]. Taken together, the evidence supports the concept that a higher percentage of LV-only fCRTp is associated with a significant incidence reduction of new-onset or sustained AF. Gurgu and Vacarescu reported favorable left atrial remodeling and improvements in diastolic function among patients with LV-only fCRTp [38,39]. These physiologic changes can likely contribute to the reduction in AF burden. In particular, Vacarescu highlighted a significant decrease in supraventricular arrhythmia burden in patients identified as left atrial responders [39].

These findings emphasize the broader physiological benefits of LV-only fCRTp, which extend beyond achieving mechanical and electrical correction of ventricular dyssynchrony to include atrial rhythm stabilization and prevention of supraventricular arrhythmia progression [70]. These observations may shift the focus back on patient selection, raising the question of whether it is necessary to exclude all patients with AF, or even those with paroxysmal episodes, who might benefit from LV-only fCRTp.

***Study limitations and future perspectives***

Given the novelty of the approach on this topic and the absence of prior publications in such a detailed and focused manner, it was challenging to anticipate the scope and consistency of the available evidence. The findings of this systematic review were not predictable, and due to expected methodological and clinical heterogeneity, a meta-analysis was not planned.

This review has some limitations, mostly determined by the analyzed studies. There was a limited number of RCTs with high statistical power and adequate blinding.

High variability in selection criteria calls for the development of a streamlined protocol for patient selection and enhanced CRT response prediction. To meet this objective, future research should focus on conducting individual patient data meta-analyses across trials to define an evidence-based profile of the ideal candidate for LV-only fCRTp.

A more systematic use of validated selection criteria, including QRS duration and morphology, along with advanced imaging markers of mechanical dyssynchrony, may help improve population homogeneity in future trials and facilitate better prediction of CRT response.

Despite constant progress, there is a persistent global underutilization of CRT devices. Even the most developed countries seem to reach certain upper thresholds regarding the ability to provide CRT implantation for eligible HFrEF patients [71]. A more cost-effective and already researched solution for some underdeveloped countries is the use of RA/LV dual-chamber CRT devices, which provide a simple and physiologic alternative for carefully selected patients. Evidence supports the importance of resynchronization as an outcome that greatly improves survival [72]. Limited resources in some clinical settings should not be prohibitive of finding solutions for achieving resynchronization, especially with a solid body of evidence regarding the safety and high efficacy of RA/LV fCRTp.

Further long-term follow-up studies on RA/LV dual-chamber CRT systems are needed to better characterize device longevity, complication risk, and cost-effectiveness in real-world patient populations.

In recent years, conduction system pacing has gained attention as a physiologic alternative to conventional BiVp [73]. However, comparative data with CRT remain limited, and a gap in evidence persists regarding their evaluation against LV-only fusion pacing. In this context, LV-only fCRTp stands out as a practical, physiology-driven approach for carefully selected patients and warrants direct comparison with other pacing modalities.

## 5. Conclusions

Despite three decades of progress, CRT implantation suffers from both underutilization and a wide variability in response to therapy. Financial constraints limit accessibility to devices, while challenges in candidate selection and optimization protocols partly drive non-response. A significant role is finally played by the overall patient profile, along with heart failure severity and the burden of comorbidities.

Careful selection and proper optimization, such as fusion strategies, are necessary to fully achieve the potential of LV-only fCRTp. This systematic review provides a comprehensive analysis of current evidence on LV-only fCRTp, focusing on refining patient selection. The marked variability in the specific selection criteria stood out in particular.

Beyond the standard guideline-based CRT indications, which are reflected by all studies, the most common specific criteria include the assessment of preserved AV conduction, a fundamental condition for achieving and maintaining effective electrical fusion between intrinsic right ventricular activation and paced LV. Furthermore, the underlying etiology and assessment of arrhythmic risk in HFrEF play a significant role in determining CRT outcomes and may even guide the choice between CRT-P and CRT-D to achieve an optimal long-term response. These additional considerations may directly affect fusion pacing or reflect substrate limitations in terms of reverse remodeling.

By systematically mapping these variables, this review consolidates current evidence and sets the stage for future research and evidence-based practice.

## Figures and Tables

**Table 4:** Specific inclusion and exclusion criteria for LV-only fCRT in studies reporting similar outcomes compared to BiVp.

No Specific Inclusion Criteria	Specific Inclusion Criteria	Specific Exclusion Criteria	
[16]	sinus rhythm [13,14,15,17,18,19,20,25]	AF	paroxysmal [17,25]
			persistent [14,17,20,25]
			permanent [14,17,19,20,25]
	PRi < 250 ms [14]	2nd- or 3rd-degree AV block [14,19,20,25]	
	2 weeks of VDD mode before randomization [13]	β-blocker therapy for ≤90 days [13]	
	LV mechanical dyssynchrony on Echo [18]	pm dependency; pacing for bradycardia [13,14,17,19,20]	
	2–8 weeks of “run-in” test before randomization [14]	patients without hemodynamic improvement during acute LV-based pacing (test response) [16]	

AF—atrial fibrillation; AV block—atrioventricular block; Echo—echocardiography; LV—left ventricle; pm dependency—pacemaker dependency; PRi—PR interval; VDD mode—pacing mode with atrial sensing and ventricular pacing; β-blocker—beta-blocker therapy.

**Table 5:** Common patient selection criteria in studies reporting superior outcomes in LV-only fCRT.

Patient Selection	Common Elements
Etiology	patients with predominantly nonischemic etiology
QRS morphology and duration	LBBB QRS duration ≥ 130–150 ms
AV conduction	preserved intrinsic AV conduction, defined as PRi ≤ 200 ms (a CRT)/1 study used PRi < 250 ms
Rhythm selection	all patients were in sinus rhythm; permanent AF was an exclusion criterion in every study
Fusion pacing algorithm	most studies used aCRT algorithm

aCRT—adaptive cardiac resynchronization therapy; AF—atrial fibrillation; AV—atrioventricular; LBBB—left bundle branch block; PRi—PR interval; QRS—QRS complex.

**Table 6:** Summary of outcomes in studies using the aCRT algorithm with high percentages of LV-only fCRTp.

Study	Definition of LV-Only fCRTp	LV-Only fCRTp (Mean) %	Results
**Martin (2012) [22]**	LV-only fCRTp ≥ 50%	64%	significant improvement in functional status and event-free survival (CCS) in 80.7% vs. 68.4% in this subgroup (*p* = 0.04)
**Birnie (2013) [23]**	LV-only fCRTp ≥ 50%	68–73%	a significantly lower risk of death or heart failure hospitalization (*p* = 0.012) in the subgroup with LV fCRTp ≥ 50%; higher rate of improvement in CCS: 80% vs. 62% compared to those with % LV fCRTp < 50% (12 months) (*p* = 0.0006)
**Burns (2017) [24]**	LV-only fCRTp ≥ 80%	>80%	greater improvements in LVEF (8.5% vs. 5.5%, *p* = 0.038) and global radial strain (6.3% vs. 4.0%, *p* = 0.046) in LV-only fCRTp compared to echo-optimized BiV pacing; 77% clinical responders in the LV-only group; (66% responders in BiV group)
**Gwag (2019) [26]**	LV-only fCRTp ≥ 50%	>97%	super-responder rate of 58.3%, compared to 36.3% in the echo-optimized BiV pacing group; no adverse clinical events in LV-only fCRTp
**Su (2021) [28]**	LV-only fCRTp with intentional non-capture RV pacing	88.7%	super-responder rate of 68.4%, compared to 36.4% in the echo-optimized BiV pacing group

CCS—clinical composite score; LVEF—left ventricular ejection fraction; LV—left ventricle; RV—right ventricle.

**Table 7:** Summary of mid- and long-term positive outcomes and complications in studies using RA/LV dual-chamber CRT devices.

Positive Effects			Complications		
Outcome	Range	References	Type	Observation	
NYHA class improvement	reported in **8/10** studies	[30,31,32,34,35,36,37,38,39]	AV block requiring upgrade	reported in 1 patient (1.8%) [36], 4 patients (5.5%) [38], and 3 patients (4%) [39]	
QRS narrowing	reported in **7/10** studies	[30,31,32,33,34,35,37]	no study reported the need for an upgrade to ICD/CRT-D during follow-up		
LVEF improvement	reported in **10/10** studies	[30,31,32,33,34,35,36,37,38,39]	**Mortality**		
**Reverse remodeling**					
LVESV reduction	reported in 6/10 studies	[31,33,35,36,37,38]	nonischemic cohorts (up to 7%)		[33,36,37,38,39]
reduction in mitral regurgitation	reported in 6/10 studies	[31,33,35,36,37,38]	ischemic patients (up to 23%)		[30,31,32,34,35]
reduction in LA volume and improvement in diastolic dysfunction profile	reported in 4/10 studies	[31,36,38,39]			
**economic benefit**	cost savings by omitting RV lead; increased device longevity	[33,35]			

AV block—atrioventricular block; CRT-D—cardiac resynchronization therapy with defibrillator; ICD—implantable cardioverter-defibrillator; LA—left atrium; LVEF—left ventricular ejection fraction; LVESV—left ventricular end-systolic volume; RV—right ventricle.

## Supplementary Materials

The following supporting information can be downloaded at https://www.mdpi.com/article/10.3390/jcm14144853/s1. S1: Search Strategy, S2: ROB Parallel-groups randomized controlled trial, S3: ROB Crossover randomized controlled trial, S4: PRISMA 2020 Checklist.

## Abbreviations

aCRT	adaptive cardiac resynchronization therapy
AF	atrial fibrillation
AV	atrioventricular
AV block	atrioventricular block
BiVp	biventricular pacing
CCS	clinical composite score
convBiVp	conventional biventricular pacing
CRT-D	cardiac resynchronization therapy with defibrillator
CRT-P	cardiac resynchronization therapy with pacemaker
DCM	dilated cardiomyopathy
DDD device	dual-chamber pacing device
DSP	desmoplakin gene
Echo	echocardiography
EKG	electrocardiogram
fCRTp	cardiac resynchronization therapy with fusion pacing
FOI	fusion-optimized interval
FLNC	filamin C gene
FU	follow-up
HF	heart failure
HR	hazard ratio
HFrEF	heart failure with reduced ejection fraction
ICD	implantable cardioverter-defibrillator
PRi	PR interval
LA	left atrium
LMNA	laminin A/C gene
LV	left ventriclel
LVEDD	left ventricular end-diastolic diameter
LVEDV	left ventricular end-diastolic volume
LGE	late gadolinium enhancement
LVEF	left ventricular ejection fraction
LVESD	left ventricular end-systolic diameter
LVESV	left ventricular end-systolic volume
MR	mitral regurgitation
MRA	mitral regurgitation area
MRI	magnetic resonance imaging
NYHA	New York Heart Association
PASP	pulmonary artery systolic pressure
pm dependency	pacemaker dependency
PQ interval/PRi	PR intervalPR interval
QRSd	QRS duration
RA	right atrium
RAAVD	right atrial-based AV delay optimization
RV	right ventricle
SCD	sudden cardiac death
SR	sinus rhythm/super-responder (depending on context)
VDD	pacing mode with atrial sensing and ventricular pacing
Vp	ventricular pacing
VV = 0	interventricular pacing delay (simultaneous BiV pacing)
6MWT	6 min walk test
VO_2_	oxygen consumption
