# Supplementary material for: Refining Patient Selection Criteria for LV-Only Fusion Pacing in Cardiac Resynchronization Therapy: A Systematic Review
Source: J Clin Med. 2025 Jul 8;14(14):4853. doi: 10.3390/jcm14144853 (PMC12294858; doi:10.3390/jcm14144853)
Supplement: Supplementary file 1 [file jcm-14-04853-s001.zip › jcm-3717449-supplementary.pdf]

S1. Search Strategy

Query strings were constructed using combinations of keywords and boolean operators to identify relevant studies in MEDLINE via PubMed and the Cochrane Library. The process involved two main blocks: (1) general CRT-related terms (*e.g.*, "cardiac resynchronization" or "biventricular pacing"), and (2) terms related to LV-only fusion pacing (*e.g.*, "LV-only", "left ventricular pacing", "univentricular pacing"). These blocks were then combined using the AND operator to isolate studies addressing both concepts. Variations of the strategy were adapted to the syntax requirements of each database, using truncation symbols (e.g., *) and proximity operators (e.g., NEXT) where applicable. The number of results retrieved at each stage is also reported for transparency. Full search strings are detailed in the table below.

Cochrane library query string

Line	Search Query	Results
#1	cardiac resynchronization OR biventricular pacing	2404
#2	fusion OR "LV only" OR (left NEXT ventric* NEXT pacing) OR "LV pacing" OR (left NEXT ventric* NEXT only) OR (univentricular NEXT pac*) OR "left univentricular" OR LUV OR "LV CRT"	11967
#3	#1 AND #2	237

PubMed query string

Line	Search Terms	Results
#1	cardiac resynchroni* OR biventricular pac*	12998
#2	fusion OR "LV only" OR "left ventric* pacing" OR "LV pacing" OR "left ventric* only" OR "univentricular pac*" OR "left univentricular" OR LUV OR "LV CRT"	382901
#3	#1 AND #2	1046

S2. ROB Parallel-groups randomized controlled trial

	Randomization process	Deviations from intended interventions	Mising outcome data	Measurement of the outcome	Selection of the reported result	Overall Bias
Assignment to intervention (the 'intention-to-treat' effect)						
Total number of study = 8						
Low risk	50	37,5	100	25	25	37,5
Some concerns	50	62,5	0	62,5	50	37,5
High risk	0	0	0	12,5	25	25

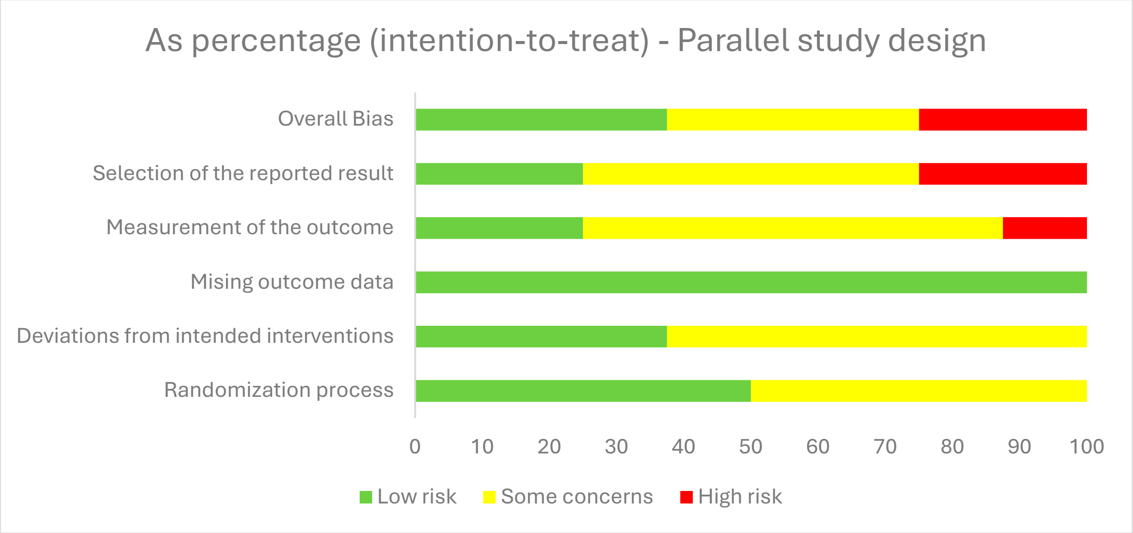

S3. ROB Crossover randomized controlled trial

	Randomization process	Bias arising from period and carryover effects	Deviations from intended interventions	Mising outcome data	Measurement of the outcome	Selection of the reported result	Overall Bias
Assignment to intervention (the 'intention-to-treat' effect)							
Total number of study = 3							
Low risk	50	0	75	75	75	25	0
Some concerns	50	75	25	25	25	50	75
High risk	0	25	0	0	0	25	25

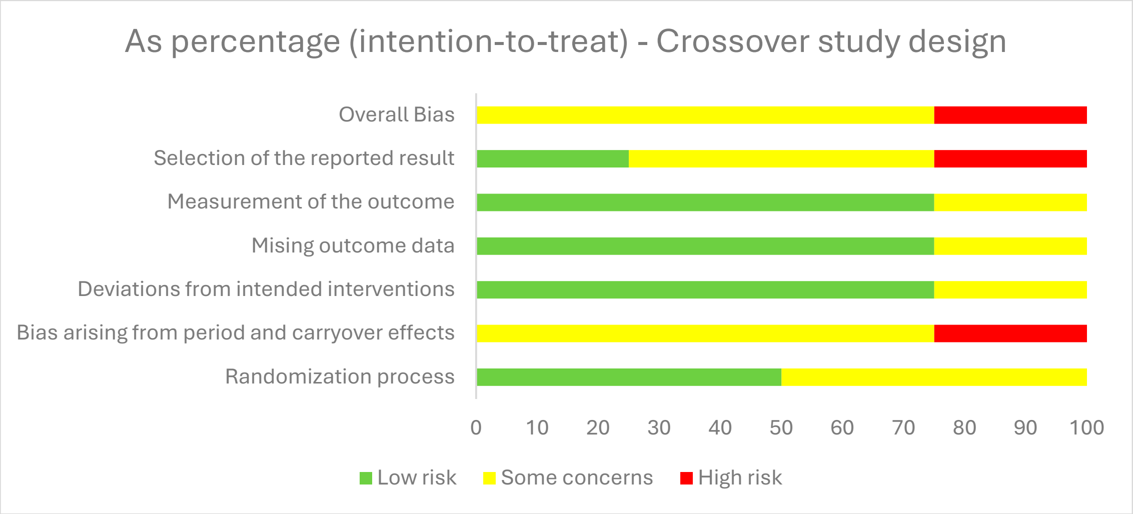

S4. PRISMA 2020 Checklist

Section and Topic	Item #	Checklist item	Location where item is reported	Page(s)	Line(s)
TITLE	1	Identify the report as a systematic review.	Title page	1	lines 1–2
ABSTRACT	2	See the PRISMA 2020 for Abstracts checklist.	Abstract section	1	lines 3–22
INTRODUCTION	3	Describe the rationale for the review in the context of existing knowledge.	Introduction	2	lines 23–59
INTRODUCTION	4	Provide an explicit statement of the objective(s) or question(s) the review addresses.	End of Introduction	2	lines 57–59
METHODS	5	Specify the inclusion and exclusion criteria for the review and how studies were grouped for the syntheses.	Methods – Eligibility Criteria	2	lines 62–79
METHODS	6	Specify all information sources (databases, registers, etc.) and date when each was last searched.	Methods – Information sources and search strategy	2–3	lines 80–89
METHODS	7	Present the full search strategies for all databases, registers, and websites, including filters and limits used.	Supplementary Material (S1)	Supplementary	not in main text
METHODS	8	Specify the methods used to decide study inclusion, number of reviewers, and use of automation tools.	Methods – Selection process	3	lines 90–96
METHODS	9	Specify data collection methods and number of reviewers, any use of automation tools.	Methods – Data collection process	3	lines 97–101
METHODS	10a	List and define outcome domains and data sought.	Methods – Data Items, PICO framework	3	lines 94-103
METHODS	10b	List and define other variables sought (e.g. participant and intervention characteristics).	Methods – Data Items	3	lines 102–106
METHODS	11	Methods for risk of bias assessment, including tools used and reviewers.	Methods – Risk of Bias Assessment	3–4	lines 107–113
METHODS	12	Effect measures (e.g. risk ratio, mean difference) used in synthesis or results.	Results – Tables and text synthesis	4–6	lines 114–215

METHODS	13a	Describe synthesis methods, grouping of studies, and comparing planned vs actual groups.	Methods – Synthesis Methods	4	lines 112–113
METHODS	13b	Describe data preparation for presentation/synthesis.	Methods – Synthesis Methods	4	lines 112–113
METHODS	13c	Describe tabulation or visual display methods.	Methods – Synthesis Methods	4	lines 112–113
METHODS	13d	Describe synthesis models, rationale, meta-analysis methods.	Not applicable – no meta-analysis	-	-
METHODS	13e	Describe methods to explore heterogeneity (e.g. subgroup analysis).	Results and Discussion – subgroup comparisons	6–7	lines 190–230
METHODS	13f	Describe any sensitivity analyses conducted.	Not applicable – no sensitivity analysis performed		
METHODS	14	Methods to assess risk of bias due to missing results (reporting biases).	Not explicitly addressed	-	-
METHODS	15	Methods to assess certainty (or confidence) in the body of evidence.	Not explicitly addressed	-	-
RESULTS	16a	Results of study selection (numbers identified, included, excluded), ideally using flow diagram.	Results – Figure 1: PRISMA flow diagram	4	line 114
RESULTS	16b	Cite studies that might appear to meet criteria but were excluded and why.	Results – Exclusion criteria summary	2	lines 75–79
RESULTS	17	Cite each included study and present its characteristics.	Results – Tables 1, 2, 3	5–7	lines 150–230
RESULTS	18	Present risk of bias assessments for each included study.	Appendix A, Tables A1-A3	Appendix A	see supplement
RESULTS	19	Results for each study including effect estimates and precision.	Results – Tables and narrative synthesis		
RESULTS	20a	For each synthesis, summarize characteristics and risk of bias.	Results – Summary in subchapters	5–7	lines 140–230
RESULTS	20b	Statistical synthesis results, including meta-analysis and heterogeneity.	Not applicable – no meta-analysis	-	-
RESULTS	20c	Investigations of heterogeneity causes.	Results and Discussion – subgroup analysis	6	lines 190-215
RESULTS	20d	Sensitivity analyses to assess result robustness.	Not applicable – no sensitivity analysis	-	-
RESULTS	21	Risk of bias due to missing results (reporting biases).	Not discussed	-	-
RESULTS	22	Certainty (or confidence) assessments.	Not discussed	-	-
DISCUSSION	23a	General interpretation in the context of other evidence.	Discussion – Opening section	7	lines 231–240
DISCUSSION	23b	Discuss any limitations of the evidence.	Discussion – Study quality and bias section	7–8	lines 241–258
DISCUSSION	23c	Discuss limitations of the review processes.	Discussion – Bias and study design discussion	8	lines 259–270
DISCUSSION	23d	Discuss implications for practice, policy, and future research.	Discussion – Final paragraphs	8	lines 270–285
OTHER INFORMATION	24a	Registration information.	PROSPERO: CRD420251034452	2	line 61
OTHER INFORMATION	24b	Where protocol can be accessed or state not prepared.	Methods – Stated adherence to PRISMA and PROSPERO	2	lines 60–62
OTHER INFORMATION	24c	Describe amendments from protocol.	Not described	-	-

OTHER INFORMATION	25	Sources of financial or non-financial support.	Not stated explicitly	-	-
OTHER INFORMATION	26	Competing interests of review authors.	Not stated explicitly	-	-
OTHER INFORMATION	27	Availability of data, code and materials.	Not described	-	-

49 Spadina Ave. Suite 200
Toronto ON M5V 2J1 Canada
www.biorender.com

Confirmation of Publication and Licensing Rights - Open Access

June 3rd, 2025

Subscription Type: *Individual - Academic*
Agreement number: *IG28CG4YDI*
Publisher Name: *Journal of Clinical Medicine*

Figure Title: *Graphical abstract*

Citation to Use: *Created in BioRender. Nistor, S. (2025) https://BioRender.com/ofchqxt*

To whom this may concern,

This document ("Confirmation") hereby confirms that Science Suite Inc. dba BioRender ("BioRender") has granted the following BioRender user: Samuel Nistor ("User") a BioRender Academic Publication License in accordance with BioRender's Terms of Service and Academic License Terms ("License Terms") to permit such User to do the following on the condition that all requirements in this Confirmation are met:

1) publish their Completed Graphics created in the BioRender Services containing both User Content and BioRender Content (as both are defined in the License Terms) in publications (journals, textbooks, websites, etc.); and
2) sublicense such Completed Graphics under "open access" publication sublicensing models such as CC-BY 4.0 and more restrictive models, so long as the conditions set forth herein are fully met.

Requirements of User:

1) All Completed Graphics to be published in any publication (journals, textbooks, websites, etc.) must be accompanied by the following citation either as a caption, footnote or reference for each figure that includes a Completed Graphic:
"Created in BioRender. Nistor, S. (2025) https://BioRender.com/ofchqxt ".
2) All terms of the License Terms including all Prohibited Uses are fully complied with. E.g. For Academic License Users, no commercial uses (beyond publication in journals, textbooks or websites) are permitted without obtaining or switching to a BioRender Industry Plan.
3) A Reader (defined below) may request that the User allow their figure to be a public template for Readers to view, copy, and modify the figure. It is up to the User to determine what level of access to grant.

Open-Access Journal Readers:

Open-Access journal readers ("Reader") who wish to view and/or re-use a particular Completed Graphic in an Open-Access journal subject to CC-BY sublicensing may do so by clicking on the URL link in the applicable citation for the subject Completed Graphic.

The re-use/modification options below are available after the Reader requests the User to adapt their

figure as a BioRender template and the User has granted such access.

1) View-Only/Free Plan Use: A Reader who wishes to only view the Completed Graphic may do so in the BioRender Services as either a BioRender Free Plan user or simply as a viewer. By becoming a BioRender Free Plan user, the Reader may view, modify and re-use the Completed Graphic as permitted under BioRender's Basic License Terms (e.g. personal use only, no publishing or commercial use permitted).
2) Re-Use/Publish with No Modifications: For any re-use and re-publication of a Completed Graphic with no modification(s) to the Completed Graphic made by the Reader, a Reader may do so by citing the original author using the citation noted above with the Completed Graphic. The Reader must also comply with the underlying License Terms which apply to the Completed Graphic as noted above (e.g. no commercial use for Academic License).
3) Re-Use/Publish with Modifications: For any re-use and re-publication of a Completed Graphic with a modification(s) made by the Reader, the Reader may do so by becoming a BioRender user themselves under either an Academic or Industry Plan, citing the original author using the citation noted above with the Completed Graphic and complying with the applicable License Terms.

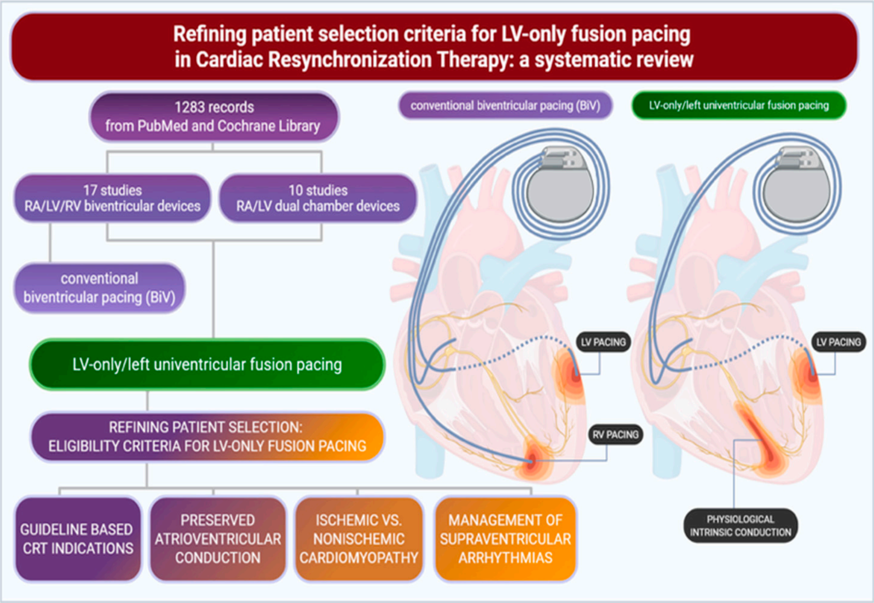

For any questions regarding this document, or other questions about publishing with BioRender, please refer to our BioRender Publication Guide, or contact BioRender Support at support@biorender.com.
